# Significant Improvement the Mechanical Properties of CoCrNi Alloy by Tailoring a Dual FCC-Phase Structure

**DOI:** 10.3390/ma13214909

**Published:** 2020-10-31

**Authors:** Hailong Yi, Mengyuan Bi, Kang Yang, Bing Zhang

**Affiliations:** 1 State Key Laboratory of Rolling and Automation, Northeastern University, Shenyang 110819, China; BiMengyuan95@163.com (M.B.); yangkang5308@163.com (K.Y.); 2 State Key Laboratory of Metastable Materials Science and Technology, Yanshan University, Qinhuangdao 066004, China

**Keywords:** SiCoCrNi alloy, microstructure, mechanical properties, FCC phase, yield strength

## Abstract

In this work, Si_x_CoCrNi (x = 0, 0.1, 0.2, 0.3, 0.4, molar ratio) alloys were designed by introducing elemental Si into CoCrNi, a medium-entropy alloy (MEA). The effects of Si addition on the microstructure and mechanical properties of the as-cast SiCoCrNi alloys were investigated. The results suggested that a novel Si-rich face-centred cubic (FCC) phase formed in the Si_0.3_CoCrNi and Si_0.4_CoCrNi alloys. The Si-rich FCC phase, which featured high hardness and was located between the dendrites of the matrix FCC phase (with higher phase fraction), significantly increased the yield strength of the Si_0.4_CoCrNi alloys five times compared with the CoCrNi alloy.

## 1. Introduction

Composed of multi-principal elements, high-entropy alloys (HEAs) and medium-entropy alloys (MEAs) have attracted wide attention in the field of materials science and the engineering community. This is owing to their excellent comprehensive mechanical properties such as high tensile strength and high plasticity, excellent high-temperature strength [1,2,3]. Among various MEAs, the CoCrNi alloy which is composed of a single face-centred cubic (FCC) phase is particularly attractive, due to its exceptional properties at cryogenic and medium temperature range over other MEAs and conventional alloys [4]. Similar to other FCC-structured MEA alloys, the CoCrNi alloy features lower yield strength and cannot meet the mechanical property requirements for engineering applications. Therefore, increasing the yield strength of CoCrNi alloy is a hot research topic [5,6].

Researchers have tried many methods to increase the strength of the CoCrNi alloy. Lu et al. reported the method of adding Nb into CoCrNi MEAs to form a Laves phase with a hexagonal close-packed structure embedded in the FCC solid solution matrix to strengthen the alloy [7]. The results indicate that the addition of Nb increases the hardness and compression yield strength of CoCrNi alloy. Wu et al. presented that the addition of W into the CoCrNi matrix enhanced the strength to an impressively high level [8]. These studies show that incorporating an element into the CoCrNi matrix can result in a good strengthening effect. Silicon is a lightweight and inexpensive element. The addition of Si into the CoCrNi matrix can reduce the alloy cost and density. In the current work, the non-metal Si was added into the CoCrNi matrix, the effects of Si addition on the microstructure evolution and mechanical properties of the as-cast CoCrNi alloys were investigated.

## 2. Materials and Methods

Alloy ingots of Si_x_CoCrNi (x = 0, 0.1, 0.2, 0.3, 0.4, molar ratio) were obtained by melting the raw materials in a non-consumable electric arc furnace under an argon atmosphere. The ingots were flipped and re-melted six times under electromagnetic stirring to ensure uniformity. The phase structure of the as-cast alloys was confirmed by X-ray diffraction (XRD, Rigaku D/MAX 2500/PC, Tokyo, Japan) with a Copper target. The characterization of microstructure details was completed by scanning electron microscopy (SEM, Hitachi S-3400N, Tokyo, Japan) and electron back-scattered diffraction (EBSD, EDAX-TSL, Amteck, Berwyn, PA, USA). Energy-dispersive X-ray spectroscopy (EDS, Amteck, Berwyn, IL, USA) analysis was performed to characterise the elemental distribution and phase composition. The EBSD samples were prepared by electro-polishing. The composition of the electrolyte was 10% perchloric acid and 90% methanol. The polishing voltage is 20 V, and the polishing time is 20 s. EBSD mapping was performed under 20 kV with a working distance of 15 mm. EBSD data were analysed using TSL-OIM Analysis 7 software package (7.2, Amteck, Berwyn, PA, USA). The microhardness of the phases in the alloys was measured using a Vickers hardness tester. The load used in the test was 25 gf and the dwell time was 10 s. The average of 10 tests was taken as the hardness value. A room-temperature uniaxial compression test was conducted on an Instron 5982 testing machine (INSTRON, Norwood, MA, USA) to identify the yield strength of the alloys. Samples for the compression tests were cylinders with an original dimension of Ф3 mm × 2 mm. The initial engineering strain rate was 0.3 × 10^−2^ s^−1^. At least three samples were tested for repeatability, and the average value was recorded.

## 3. Results and Discussion

### 3.1. Microstructure

**Figure 1:**
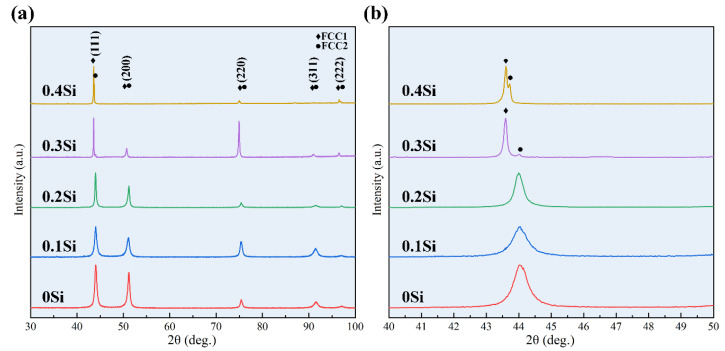
(**a**) X-ray diffraction (XRD) patterns of the as-cast Si_x_CoCrNi (x = 0, 0.1, 0.2, 0.3, 0.4) alloys and (**b**) partially enlarged view of (**a**).

Figure 1a shows the XRD patterns of the as-cast Si_x_CoCrNi (x = 0, 0.1, 0.2, 0.3, 0.4) alloys. Figure 1b is the enlarged view of Figure 1a from 40° to 50°. According to the XRD indexing results, the CoCrNi, Si_0.1_CoCrNi and Si_0.2_CoCrNi alloys consisted of a single FCC phase. With the increase in the Si content, the diffraction peaks of Si_0.3_CoCrNi and Si_0.4_CoCrNi alloys split. Besides the matrix FCC phase, another new FCC phase formed in these two alloys.

This result is different from the research result of other researchers [9,10]. They only obtained a single FCC phase structure in the heat-treated NiCoCrSi_0.3_ alloy. This should be attributed to different alloy processing methods. In Reference [9], the NiCoCrSi_0.3_ alloy underwent a series of complex treatment processes, namely, homogenization annealing, cold rolling, post-rolling annealing and water quenching. In Reference [10], the CrCoNiSi_0.3_ alloy also underwent a series of treatments similar to those in Reference [9], namely homogenization annealing, cold rolling, recrystallization annealing and water quenching. The main difference between the heat treatment process of Reference [9] and Reference [10] is that the annealing temperature before water quenching is different. The annealing temperatures for the two different treatments are 800 and 900 °C, respectively. After annealing and water quenching at 800 °C, besides the FCC phase, there are compounds precipitated in the alloy, while after annealing and water quenching at 900 °C, there is only a single FCC phase. This indicates that silicon can be completely dissolved in the alloy when the temperature is 900 °C or higher. While in this study, the Si_0.3_CoCrNi alloy is an as-cast sample, and the solidification process of the sample is completed in a water-cooled copper crucible. The as-cast Si_0.3_CoCrNi alloy is directly cooled from liquid. Thus, it can be considered that the as-cast Si_0.3_CoCrNi alloy is cooled from a completely solid solution state. The cooling rate of the water-cooled copper crucible is lower than the cooling rate of water quenching, but is much higher than the cooling rate of the equilibrium transformation. Therefore, dual FCC phases formed in the as-cast Si_0.3_CoCrNi alloy rather than a single FCC phase or intermetallic compound plus FCC phase. Some researchers also found that metastable solid solutions can form preferably over intermetallic compounds in cast high-entropy alloys or multi-component alloys with equi- or nearly equi-atomic compositions [11]. This is consistent with the current study.

**Figure 2:**
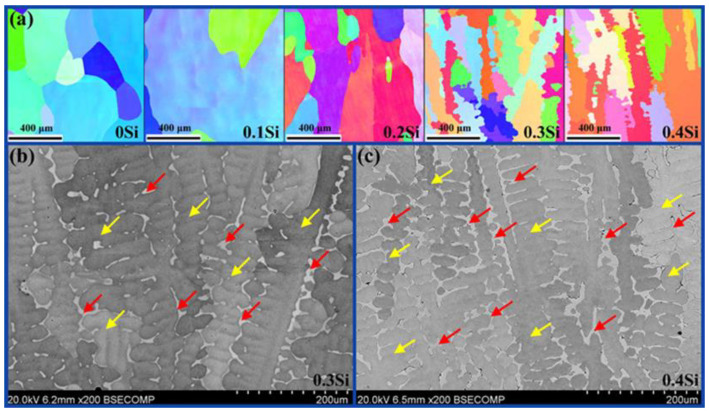
(**a**) Inverse pole figure (IPF) maps of the as-cast Si_x_CoCrNi (x = 0, 0.1, 0.2, 0.3, 0.4) alloys; SEM images of the as-cast, (**b**) Si_0.3_CoCrNi and (**c**) Si_0.4_CoCrNi alloys.

Figure 2a is the inverse pole figure (IPF) maps of the as-cast Si_x_CoCrNi (x = 0, 0.1, 0.2, 0.3, 0.4) alloys. It can be seen that the microstructures of the as-cast alloys changed with the increase in the Si content. The IPF maps indicate that the CoCrNi, Si_0.1_CoCrNi and Si_0.2_CoCrNi alloys consisted of coarse columnar grains. The Si_0.3_CoCrNi and Si_0.4_CoCrNi alloys consisted of dendrites. In addition, a significant refinement occurred in the alloys. Figure 2b,c show the backscattered electron (BSE) signal source SEM images of the Si_0.3_CoCrNi and Si_0.4_CoCrNi alloys. It can be drawn from the BSE images that the new FCC phase (marked with red arrows) was located between the matrix dendrites (marked with yellow arrows). Furthermore, the volume fraction and grain size of the new FCC phase in the Si_0.4_CoCrNi alloy increased to a greater extent than those in the Si_0.3_CoCrNi alloy. These SEM results are consistent with the IPF map results. Image J software was used to perform statistical analysis on SEM photos to determine the phase fraction and grain size of the new FCC phase (FCC2) in Si_0.3_CoCrNi and Si_0.4_CoCrNi alloys. According to the statistical results, the phase fraction of the new FCC phase (FCC2) in Si_0.3_CoCrNi and Si_0.4_CoCrNi alloys are 7.8% and 17.8%, respectively, while the grain sizes in these two alloys are 136.2 and 241.9 μm^2^, respectively.

**Figure 3:**
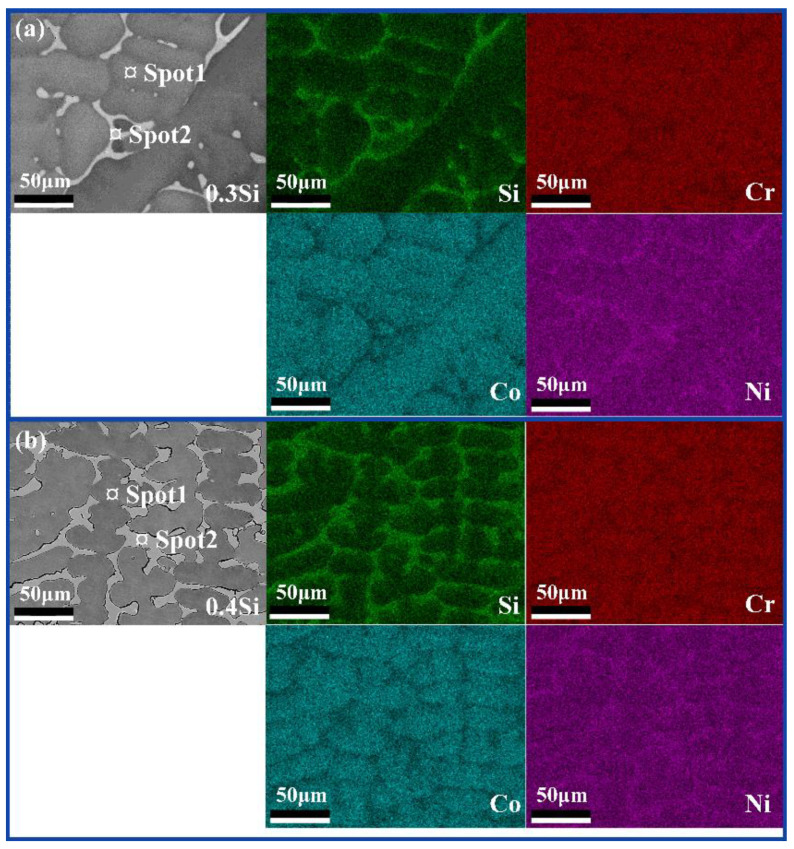
Elements mapping maps of the as-cast (**a**) Si_0.3_CoCrNi and (**b**) Si_0.4_CoCrNi alloys.

**Figure 4:**
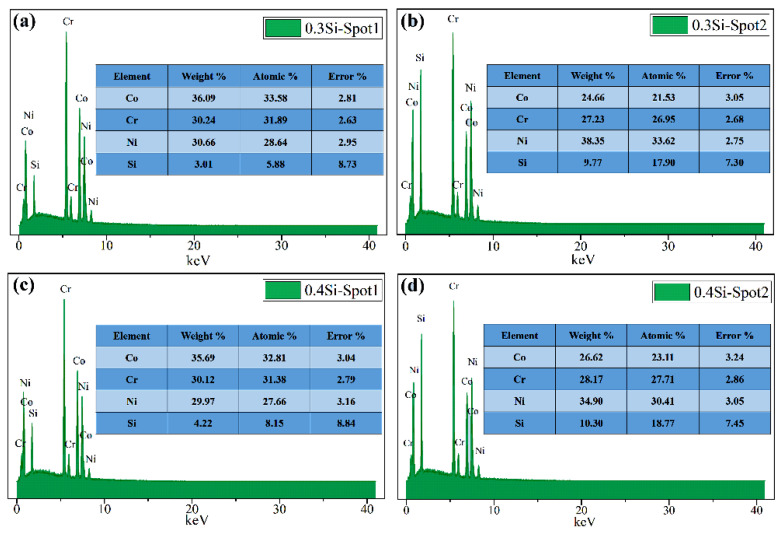
Energy-dispersive X-ray spectroscopy (EDS) point analysis results of the spots as marked in Figure 3. (**a**) 0.3Si-spot1; (**b**) 0.3Si-spot2; (**c**) 0.4Si-spot1 and (**d**) 0.4Si-spot2.

Figure 3 and Figure 4 show the EDS results of the Si_0.3_CoCrNi and Si_0.4_CoCrNi alloys. According to the mapping results, significant differences existed between the elemental distributions of two FCC phases. In the Si_0.3_CoCrNi alloy, the Si, Co, Cr and Ni contents of the matrix FCC phase were 5.88%, 33.58%, 31.89% and 28.64% (at.%), respectively. The Si, Co, Cr and Ni of the new FCC phase were 17.9%, 26.95%, 21.53% and 33.62% (at.%), respectively. However, in the Si_0.4_CoCrNi alloy, the contents of the above elements in the matrix FCC phase were 8.15%, 32.81%, 31.38% and 27.66% (at.%), respectively, and those in the new FCC phase were 18.77%, 23.11%, 27.71% and 30.41% (at.%), respectively. The Co, Cr and Ni contents of the matrix FCC phase in the Si_0.3_CoCrNi and Si_0.4_CoCrNi alloys were near-equiatomic. In the new FCC phase, the Si content increased and the Co contents significantly decreased.

### 3.2. Mechanical Properties

**Figure 5:**
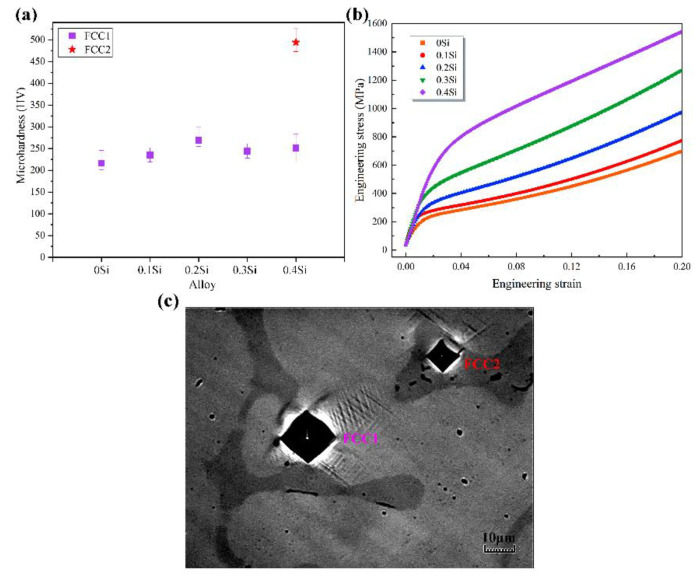
(**a**) Microhardness, (**b**) compression curves of the as-cast Si_x_CoCrNi (x = 0, 0.1, 0.2, 0.3, 0.4) alloys and (**c**) typical Vickers hardness indentation morphologies of the as-cast Si_0.4_CoCrNi alloy.

Figure 5a shows the microhardness test results of the as-cast SixCoCrNi (x = 0, 0.1, 0.2, 0.3, 0.4) alloys. The hardness of the matrix FCC phase in the Si_x_CoCrNi (x = 0, 0.1, 0.2, 0.3, 0.4) alloys showed only small variations with the change in the Si content. This indicates that the refinement and a small increase in the Si content did not significantly strengthen the matrix FCC phase. However, the new FCC phase exhibited a sharp hardness increase compared with the matrix FCC phase in the Si_0.4_CoCrNi alloy; this is probably due to the substantial increase in the Si content and the small grain size of the new FCC phase. Figure 5b shows the compression curves of the as-cast Si_x_CoCrNi (x = 0, 0.1, 0.2, 0.3, 0.4) alloys. The yield strengths of the as-cast Si_x_CoCrNi (x = 0, 0.1, 0.2, 0.3, 0.4) alloys were 131, 208, 267, 342 and 655 MPa, respectively. Fractures did not occur during the compression tests of the CoCrNi, Si_0.1_CoCrNi and Si_0.2_CoCrNi alloys. Both the fracture elongations of the Si_0.3_CoCrNi and Si_0.4_CoCrNi alloys exceeded 30%. This indicated that by adding a small amount of silicon to the CoCrNi MEA, the compressive yield strength of the CoCrNi MEA can be increased by five times without complex heat treatment.

With the increase in the Si content from 0 at.% to 9.1 at.% (Si_0.3_CoCrNi), the yield strength of the alloys increased slowly. However, the Si_0.4_CoCrNi alloy featured a dramatic enhancement in yield strength compared with the Si_0.3_CoCrNi alloy. Figure 5c shows the typical Vickers hardness indentation morphologies of the as-cast Si_0.4_CoCrNi alloy. From the image, it can be seen that the new FCC phase (FCC2) has a much higher hardness than the matrix phase. According to the hardness test results, the enhancement was not caused by the solid solution of Si in the matrix FCC phase (FCC1); it was mainly caused by the formation of the new FCC phase (FCC2). The strength of Si_0.3_CoCrNi and Si_0.4_CoCrNi alloys composed of a dual FCC-phase structure can be expressed by the rule of the mixture [12,13]:

σy = σ_FCC1_·*V*_FCC1_ + σ_FCC2_·*V*_FCC2_ (1)

In the formula, σ and *V* stand for the yield strength and the volume proportion of each phase. It can be seen from the above formula that the formation of the new FCC phase with higher hardness can significantly increase the yield strength of the alloy. The result showed that by designing a novel MEA with a dual-phase FCC structure, the yield strength of the CoCrNi alloy can be greatly improved. This research provided a new idea for improving the comprehensive mechanical properties of CoCrNi medium-entropy alloy.

## 4. Conclusions

In summary, the addition of Si into the CoCrNi alloys significantly changed the microstructure, phase and mechanical property of the as-cast alloys. The as-cast CoCrNi, Si_0.1_CoCrNi and Si_0.2_CoCrNi alloys consisted of coarse columnar grains with a single FCC phase. The as-cast Si_0.3_CoCrNi and Si_0.4_CoCrNi alloys consisted of dendrites with a dual FCC-phase structure. The Si-rich new FCC phase, which had high hardness and was located between the dendrites of the matrix FCC phase, significantly increased the yield strength of the Si_0.4_CoCrNi alloy.

## References

[1] Moravcik I., Gouvea L., Cupera J., Dlouhy I. (2018). Preparation and properties of medium entropy CoCrNi/boride metal matrix composite. J. Alloys Compd..

[2] Sathiyamoorthi P., Moon J., Bae J.W., Asghari-Rad P., Kim H.S. (2019). Superior cryogenic tensile properties of ultrafine-grained CoCrNi medium-entropy alloy produced by high-pressure torsion and annealing. Scr. Mater..

[3] Vilémová M., Hadraba H., Weiss Z., Lukáč F., Csáki Š., Chlup Z., Matějíček J., Chráska T. (2020). Phase, Composition and Structure Changes of CoCrNi-Based Concentrated Alloys Resulting from High Temperature Oxidation. Materials.

[4] Sathiyamoorthi P., Bae J.W., Asghari-Rad P., Park J.M., Kim J.G., Kim H.S. (2018). Effect of Annealing on Microstructure and Tensile Behavior of CoCrNi Medium Entropy Alloy Processed by High-Pressure Torsion. Entropy.

[5] Wang J., Yang H., Huang H., Ruan J., Ji S. (2019). Microstructure and mechanical properties of SiC whisker reinforced CoCrNi medium entropy alloys. Mater. Lett..

[6] Lee D., Jeong H.-U., Lee K.-H., Jeon J.B., Park N. (2019). Precipitation and grain-boundary strengthening of Al-added CoCrNi medium-entropy alloys. Mater. Lett..

[7] Lu W., Luo X., Yang Y., Huang B. (2019). Effects of Nb additions on structure and mechanical properties evolution of CoCrNi medium-entropy alloy. Mater. Express.

[8] Wu Z., Guo W., Jin K., Poplawsky J.D., Gao Y., Bei H. (2018). Enhanced strength and ductility of a tungsten-doped CoCrNi medium-entropy alloy. J. Mater. Res..

[9] Liu S., Lin W., Zhao Y., Chen D., Yeli G., He F., Zhao S., Kai J.-j. (2020). Effect of silicon addition on the microstructures, mechanical properties and helium irradiation resistance of NiCoCr-based medium-entropy alloys. J. Alloys Compd..

[10] Chang H., Zhang T.W., Ma S.G., Zhao D., Xiong R.L., Wang T., Li Z.Q., Wang Z.H. (2021). Novel Si-added CrCoNi medium entropy alloys achieving the breakthrough of strength-ductility trade-off. Mater. Des..

[11] Guo S., Hu Q., Ng C., Liu C.T. (2013). More than entropy in high-entropy alloys: Forming solid solutions or amorphous phase. Intermetallics.

[12] Cheng P., Zhao Y., Xu X., Wang S., Sun Y., Hou H. (2020). Microstructural evolution and mechanical properties of Al0.3CoCrFeNiSix high-entropy alloys containing coherent nanometer-scaled precipitates. Mater. Sci. Eng. A.

[13] He J.Y., Wang H., Huang H.L., Xu X.D., Chen M.W., Wu Y., Liu X.J., Nieh T.G., An K., Lu Z.P. (2016). A precipitation-hardened high-entropy alloy with outstanding tensile properties. Acta Mater..

